# Derivation and validation of a simple clinical bedside score (ATLAS) for *Clostridium difficile* infection which predicts response to therapy

**DOI:** 10.1186/1471-2334-13-148

**Published:** 2013-03-25

**Authors:** Mark A Miller, Thomas Louie, Kathleen Mullane, Karl Weiss, Arnold Lentnek, Yoav Golan, Yin Kean, Pam Sears

**Affiliations:** 1 Division of Infectious Diseases, Jewish General Hospital, 3755 Cote-Ste-Catherine Rd, Montreal, QC, Canada; 2 University of Calgary, Calgary, AB, Canada; 3 University of Chicago, Chicago, IL, USA; 4 Hopital Maisonneuve-Rosemont, Montreal, QC, Canada; 5 Wellstar Infectious Disease, Marietta, GA, USA; 6 Tufts Medical Center, Boston, MA, USA; 7 Optimer Pharmaceutical, Inc, San Diego, CA, USA

## Abstract

**Background:**

Clostridium difficile infection (CDI) continues to be a frequent and potentially severe infection. There is currently no validated clinical tool for use at the time of CDI diagnosis to categorize patients in order to predict response to therapy.

**Methods:**

Six clinical and laboratory variables, measured at the time of CDI diagnosis, were combined in order to assess their correlation with treatment response in a large CDI clinical trial database (derivation cohort). The final categorization scheme was chosen in order to maximize the number of categories (discrimination) while maintaining a high correlation with clinical cure assessed two days after the end of therapy. Validation of the derived scoring scheme was done on a second large CDI clinical trial database (validation cohort). A third comparison was done on the two pooled databases (pooled cohort).

**Results:**

In the derivation cohort, the best discrimination and correlation with cure was seen with a five-component ATLAS score (age, treatment with systemic antibiotics, leukocyte count, albumin and serum creatinine as a measure of renal function), which divided CDI patients into 11 groups (scores of 0 to 10 inclusive) and was highly correlated with treatment outcome (R^2^=0.95; P<0.001). This scheme showed excellent prediction of cure in the validation cohort (overall Kappa=95.2%; P<0.0001), as well as in the pooled cohort, regardless of treatment (fidaxomicin or vancomycin).

**Conclusions:**

A combination of five simple and commonly available clinical and laboratory variables measured at the time of CDI diagnosis, combined into a scoring system (ATLAS), are able to accurately predict treatment response to CDI therapy. The ATLAS scoring system may be useful in stratifying CDI patients so that appropriate therapies can be chosen to maximize cure rates, as well as for categorization of patients in CDI therapeutic studies in order allow comparisons of patient groups.

## Background

*Clostridium difficile* infection (CDI) has emerged during the last decade as a serious and increasingly common healthcare-associated infection [1]. The emergence of hypervirulent strains has resulted in elevated rates of CDI-related complications (e.g. colectomy, need for intensive care) and increased mortality, especially among the elderly [2,3]. Newer treatment options, such as novel antibacterials [4], immune modulators [5] and immunotherapeutics [6], have led to a recent expansion in the number of clinical trials involving subjects with this serious infection. Despite more than 3 decades of research involving this condition, a validated “severity scale” has yet to be developed which correlates with treatment response, which is predictive of severe outcomes (i.e. colectomy, need for intensive care, or attributable mortality) or which predicts CDI recurrence. Although several predictive scoring systems and clinical variables have been described in limited CDI populations or case series, none have been validated on large CDI databases [7-16]. While the choice of therapy for malignant neoplasms and subjects with sepsis is often based on validated criteria which consist of both patient and disease-related variables in order to maximize treatment response [17,18], no such validated scheme exists for CDI. The absence of such a categorization system means that the choice of therapies for a particular patient is often not evidence-based, and clinical trials investigating CDI outcomes may be comparing dissimilar patient populations. Recent guidelines which have put forward “severity categories” for CDI have not validated these categorizations and their correlation with treatment outcome, disease outcome and CDI recurrence are unknown [19,20]. We have used 2 large clinical therapeutic trials for treating CDI in order to derive and then validate a categorization system to discriminate among CDI patients and correlate the grouping with treatment response.

## Methods

Two large databases, which were derived while conducting therapeutic trials that compared fidaxomicin and vancomycin for the treatment of CDI, were used for the present analyses [4,21]. The clinical and trial details for the two identical CDI therapy studies are described elsewhere [4]. Briefly, 10 days of therapy with either vancomycin or fidaxomicin was administered to CDI patients. The first trial (“003”) enrolled patients in the United States and Canada; the second trial (“004”) enrolled patients in those two countries as well as in Europe. The response to treatment was assessed two days following the last day of therapy. Patients considered as a “cure” were then followed for an additional 28 days to evaluate them for a CDI recurrence. This present analyses used all patients included in each of the respective trials if they had a confirmed diagnosis of CDI and received at least 1 dose of study medication (“modified intent to treat” group; mITT). Since the vancomycin and fidaxomicin arms had nearly identical cure rates [4], all patients in each study were combined into a single group regardless of the therapy they were randomized to receive. The mITT group of patients consisted of 596 individuals in the 003 study, 509 individuals in the 004 study, and a total of 1105 subjects in both combined studies. All subjects in both studies gave informed consent which also allowed for secondary analyses of the databases such as in the present investigation. No additional form of ethical approval was required to do this subgroup risk analysis, as the original ethical approval for the trial covered such analyses. The study sponsor permitted the authors to access the trial data for this analysis; the dataset used was preexisting, de-identified and required no further collection of data from patients.

The six clinical and laboratory parameters used in the analyses were chosen for their ready availability, their ease of calculation, prior correlation with CDI outcome in case series [7-16], and the fact that they had been collected and were available in the two CDI clinical trials of interest. The six parameters, measured on the day of entry into the study (i.e. within 48 hours of a positive *C. difficile* toxin assay) were: age (in years) [Ag], treatment with systemic antibiotics (which occurred on one or more days of CDI therapy) [Tr], temperature (in degrees Celsius) [Te], total leukocyte count [L], serum albumin [Al], and serum creatinine as a measure of renal function [S]. A logistic regression model was created using “cure” as the dependant variable and the six clinical/lab parameters as the independent variables. Due to high co-correlation of some of the six independent variables (i.e. age, albumin and serum creatinine showing high Pearson correlation coefficients), it was decided instead to use these indices in a clinical score. Scores of 0, 1 or 2 were assigned to each parameter value, based on their relative importance as seen in previously published analyses (Table 1). All possible combinations of these six parameters were assessed for their correlation with cure at end of therapy. Correlation was assessed using linear regression, with a forced value of 100 on the y axis for a score of 0 on the x axis, in order to make the ensuing regression formula clinically meaningful. Statistical analyses were performed with SPSS software.

**Table 1:** Clinical and laboratory variables, along with their respective values and points, for determining the optimal scoring system which correlates with cure after CDI therapy

**Parameter**	**0 points**	**1 point**	**2 points**
Age	< 60 years	60 – 79 years	≥ 80 years
Treatment with systemic antibiotics during CDI therapy (≥ 1 day)	No	---------	Yes
Temperature	≤ 37.5°C	37.6 – 38.5°C	≥ 38.6°C
Leukocyte count (total)	< 16,000	16,000 – 25,000	> 25,000
Albumin (serum)	> 35 g/L	26 – 35 g/L	≤ 25 g/L
Serum creatinine (as a measure of renal function)	≤ 120 μmol/L	121 – 179 μmol/L	≥ 180 μmol/L

During the first step of this analysis, the derivation process, the 003 trial database was used to test the combinations for their correlation with treatment response.

The optimal combination in this derivation analysis, to be used for the validation analysis, was considered to be the combination which met the following criteria:

i. Correlation with cure having an R^2^ value ≥ 0.9

ii. Correlation with cure having a P value ≤ 0.01

If multiple combinations met the above 2 conditions, the combination consisting of the largest number of variables was considered as the optimal combination, since this scheme would be able to categorize the patients into the largest number of distinct groups (i.e. highest discriminative ability and category utility) [22].

During the second step of this analysis, the validation process, the predicted cure rate from the optimum combination chosen in step one (using the derived regression formula) was compared to the actual CDI cure rate as seen in the 004 clinical trial database. This was done by means of Kappa statistics of the cure rate for each score category.

During a final step of this analysis, the two clinical studies (003 and 004) were pooled and the optimum combination which had been derived in the first step was used to compare the predicted and actual cure rates for this entire cohort, again using a Chi-Square analysis for each score group.

## Results

Step 1. Derivation of the optimal categorization scoring system.

All of the possible combinations and permutations of the six chosen variables are listed in Table 2, along with their respective correlations with treatment response in the first (003) clinical trial, as demonstrated by the respective R^2^ and P values. The 12 combinations which each demonstrated an R^2^ value of ≥ 0.9 with a P value of ≤ 0.01 were:

1) Age, albumin

2) Temperature, albumin

3) Age, leukocyte count

4) Age, treatment with systemic antibiotics, leukocyte count

5) Age, temperature, serum creatinine

6) Age, temperature, leukocyte count

7) Temperature, albumin, leukocyte count

8) Age, treatment with systemic antibiotics, temperature, serum creatinine

9) Age, temperature, albumin, serum creatinine

10) Age, temperature, leukocyte count, albumin

11) Temperature, leukocyte count, albumin, serum creatinine

12) Age, treatment with systemic antibiotics, leukocyte count, albumin, serum creatinine

**Table 2:** **All possible combinations of the six clinical and laboratory variables, along with the respective linear correlation coefficient (R**^**2**^**) and its significance (P value), when correlated with CDI cure for patients in the 003 trial database with available values for all analyzed variables; n=515**

	**R**^**2**^	**P value**		**R**^**2**^	**P value**
Single variables			Four variables		
Ag	0.91	0.195	Ag, Tr, Te, S *	0.92	<0.001
Tr	1.0	N/A	Ag, Tr, Te, Al	0.79	0.001
Te	0.95	0.147	Ag, Tr, Te, L	0.89	<0.001
L	1.0	0.025	Ag, Tr, Al, S	0.82	<0.001
Al	0.99	0.070	Ag, Tr, L, S	0.25	0.175
S	0.47	0.518	Ag, Tr, L, Al	0.75	0.001
			Ag, Te, Al, S *	0.95	<0.001
Two variables			Ag, Te, L, S	0.78	0.008
Ag, Tr	0.78	0.048	Ag, Te, L, Al *	0.91	<0.001
Ag, Te	0.84	0.011	Ag, L, Al, S	0.87	0.001
Ag, S	0.49	0.191	Tr, Te, Al, S	0.79	0.001
Ag, Al *	0.97	0.003	Tr, Te, L, S	0.82	0.001
Ag, L	0.68	0.087	Tr, Te, L, Al	0.86	0.003
Tr, Te	0.79	0.044	Tr, L, Al, S	0.65	0.016
Tr, S	0.70	0.077	Te, L, Al, S *	0.91	<0.001
Tr, Al	0.84	0.030			
Tr, L	0.26	0.375	Five variables		
Te, S	0.06	0.702	Ag, Tr, Te, Al, S	0.80	<0.001
Te, Al *	0.92	0.002	Ag, Tr, Te, L, S	0.82	0.002
Te, L	0.89	0.058	Ag, Tr, S, L, Al **	0.95	<0.001
Al, S	0.90	0.015	Ag, Te, L, Al, S	0.78	0.001
L, S	0.63	0.108	Ag, Tr, Te, L, Al	0.86	<0.001
L, Al *	0.94	0.006	Tr, Te, L, Al, S	0.79	0.001
Three variables			Six variables		
Ag, Tr, Te	0.85	0.003	Ag, Tr, Te, L, Al, S	0.70	0.001
Ag, Tr, S	0.86	0.003			
Ag, Tr, Al	0.86	0.003			
Ag, Tr, L *	0.96	<0.001			
Ag, Te, S *	0.98	0.001			
Ag, Te, Al	0.83	0.002			
Ag, Te, L *	0.90	0.001			
Ag, Al, S	0.86	0.002			
Ag, L, S	0.10	0.482			
Ag, L, Al	0.810	0.002			
Tr, Te, S	0.82	0.005			
Tr, Te, Al;	0.82	0.002			
Tr, Te, L	0.71	0.036			
Tr, Al, S	0.84	0.004			
Tr, L, S	0.00	0.975			
Tr, L, Al	0.49	0.081			
Te, Al, S	0.17	0.366			
Te, L, S	0.84	0.029			
Te, L, Al *	0.95	<0.001			
L, Al, S	0.79	0.003			

All combinations meeting the pre-set “acceptable” criteria (R^2^ ≥ 0.9 and P ≤ 0.01) are marked with an asterisk (*), and the single combination among this latter group which was composed of the largest number of variables is marked with a double asterisk (**) as the optimal scoring system.

Ag = age; Tr = treatment with systemic antibiotics; Te = temperature; L = leukocyte count (total); Al = serum albumin; S = serum creatinine as a measure of renal function.

CDI: *Clostridium difficile* infection.

N/A: not applicable.

**Figure 1:**
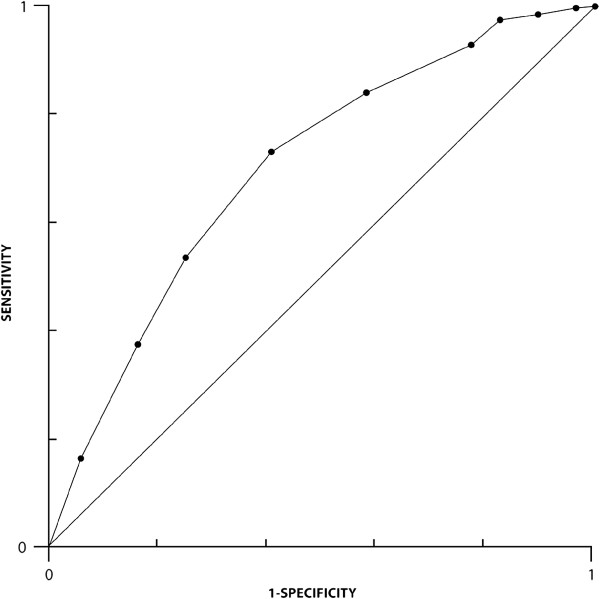
Receiver operating characteristics of the ATLAS score for predicting treatment cure (area under the curve = 0.71).

Of these 12 candidate combinations, the most discriminating (containing the largest number of variables and thereby separating the patients into the largest number of unique groups) was the combination of age (Ag), treatment with systemic antibiotics (Tr), leukocyte count (L), serum albumin (Al) and serum creatinine as a measure of renal function (S) (abbreviated herewith as ATLAS). The ATLAS combination produced a scoring system which was able to place the CDI patients into 11 unique categories (scores 0 to 10, inclusive) and this correlated with treatment cure with an R-squared value of 0.95 and a highly significant P value of < 0.001. The regression equation for this correlation was shown to be: cure rate = 100 – [5.08 × (ATLAS score)]. The receiver operating characteristics of the ATLAS score for predicting treatment cure are shown in Figure 1 (area under the curve was calculated to be 0.71).

Step 2. Validation of the categorization system from step 1.

The actual cure rates and the predicted cure rates (derived from the regression equation calculated in step 1) for each of the ATLAS categories of the CDI patients in the mITT population of the 004 clinical trial are seen in Table 3.

**Table 3:** The predicted cure rate and the actual cure rate for CDI patients in the validation database (all mITT patients in the 004 clinical trial with available values for all analyzed variables; n=452)

**ATLAS score (n**_**T**_**)**	Predicted cure rate* (%) (n_**c**_^**§**^**/n**_**T**_**)**	**Actual cure rate (%) (n**_**c**_**/n**_**T**_**)**	**Kappa**	**P value**
0 (69)	100 (69/69)	95.7 (66/69)	N/A	N/A
1 (89)	94.9 (84/89)	93.3 (83/89)	90.3%	< 0.0001
2 (68)	89.8 (61/68)	92.7 (63/68)	81.8%	< 0.0001
3 (86)	84.8 (73/86)	89.5 (77/86)	79.3%	< 0.0001
4 (53)	79.7 (42/53)	81.1 (43/53)	94.1%	< 0.0001
5 (46)	74.6 (34/46)	76.1 (35/46)	94.2%	< 0.0001
6 (21)	69.5 (15/21)	85.7 (18/21)	58.8%	0.0031
7 (8)	64.4 (5/8)	50 (4/8)	75.0%	0.0285
8 (9)	59.4 (5/9)	55.6 (5/9)	100%	0.0027
9 (3)	54.3 (2/3)	33 (1/3)	40.0%	0.3865
10 (0)	49.2 (N/A)	N/A	N/A	N/A
All scores (452)	86.3 (390/452)	87.4 (395/452)	95.2%	< 0.0001

CDI patients categorized by the ATLAS scoring system, using the regression formula* derived from the ATLAS system applied to the 003 clinical trial. Testing the reliability of the predicted cure rate compared to the actual cure rate among individual ATLAS groups and for the entire population was done using Kappa statistics.

* Predicted cure rate = 100–5.08 x (ATLAS score).

n_c_ : number of subjects cured in the category.

n_T_ : total number of subjects in the category.

^§^n_c_ values for the predicted column were rounded to the nearest whole number.

N/A: not applicable NS: not significant.

The ATLAS categorization scheme was able to very closely (overall Kappa=95.2%; P<0.0001) predict the actual cure rate for these patients in every score category.

Step 3. Capacity of the ATLAS score to predict cure for the pooled CDI patient databases, and by treatment allocation.

In order to assess how the ATLAS scoring system would perform when all patients in both trials were placed into a single database, this categorization scheme was used to compare the predicted cure rates and the actual cure rates for all mITT patients, as well as for the two sub-groups of patients as determined by treatment allocation (i.e. fidaxomicin or vancomycin). The results of these analyses can be seen in Table 4. Again, excellent predictive ability of the ATLAS system is seen for the entire database and for each of the two assigned treatment groups.

**Table 4:** The predicted and actual cure rates for CDI patients in the combined 003 and 004 trial databases (mITT patients who have available values for all analyzed variables), and also categorized by treatment assignment on entry into the studies (fidaxomicin or vancomycin)

	**All patients (n=967)**		**Vancomycin patients (n=494)**		**Fidaxomicin patients (n=473)**	
**ATLAS score**	Predicted cure rate* (%) (n_**c**_**/n**_**T**_**)**	**Actual cure rate (%) (n**_**c**_**/n**_**T**_**)**	Predicted cure rate* (%) (n_**c**_**/n**_**T**_**)**	**Actual cure rate (%) (n**_**c**_**/n**_**T**_**)**	Predicted cure rate* (%) (n_**c**_**/n**_**T**_**)**	**Actual cure rate (%) (n**_**c**_**/n**_**T**_**)**
0	100 (161/161)	96.3 (155/161)	100 (75/75)	93.3 (70/75)	100 (86/86)	98.8 (85/86)
1	94.9 (160/169)	93.5 (158/169)	94.9 (84/89)	93.3 (83/89)	94.9 (76/80)	93.8 (75/80)
2	89.8 (144/160)	93.1 (149/160)	89.8 (72/80)	93.8 (75/80)	89.8 (72/80)	92.5 (74/80)
3	84.8 (146/172)	87.2 (150/172)	84.8 (72/85)	87.1 (74/85)	84.8 (74/87)	87.4 (76/87)
4	79.7 (99/124)	78.2 (97/124)	79.7 (58/73)	76.7 (56/73)	79.7 (41/51)	80.4 (41/51)
5	74.6 (68/91)	81.3 (74/91)	74.6 (32/43)	76.7 (33/43)	74.6 (36/48)	85.4 (41/48)
6	69.5 (40/58)	74.1 (43/58)	69.5 (19/28)	78.6 (22/28)	69.5 (21/30)	70 (21/30)
7	64.4 (10/16)	62.5 (10/16)	64.4 (8/12)	58.3 (7/12)	64.4 (3/4)	75 (3/4)
8	59.4 (7/11)	54.6 (6/11)	59.4 (4/7)	57.1 (4/7)	59.4 (2/4)	50 (2/4)
9	54.3 (3/5)	40 (2/5)	54.3 (1/2)	0 (0/2)	54.3 (2/3)	66.7 (2/3)
10	49.2 (N/A)	N/A	49.2 (N/A)	N/A	49.2 (N/A)	N/A
All scores	86.7 (838/967)	87.3 (844/967)	86.0 (425/494)	85.8 (424/494)	87.3 (413/473)	88.8 (420/473)

CDI patients categorized by the ATLAS scoring system, using the regression formula* derived from the ATLAS system applied to the 003 clinical trial.

* Predicted cure rate = 100–5.08 x (ATLAS score).

n_c_: number of subjects cured in the category n_T_ : total number of subjects in the category N/A: not applicable.

n_c_ values for the predicted column were rounded to the nearest whole number.

## Discussion

The current classifications of “mild”, “moderate”, “severe”, and “fulminant” CDI are unvalidated, subjective, and have not yet been shown to be clinically useful. An easy to use, objective, clinically relevant and validated system of categorizing CDI patients is needed in order to choose among the growing number of available therapies, to decide which patients might benefit from adjunctive therapies, to facilitate communication among healthcare providers, to prognosticate the outcome of therapy, and to categorize patients for CDI intervention trials.

Two large CDI clinical trial databases, the fidaxomicin/vancomycin comparative trials, were used to derive and then to validate a CDI patient categorization scheme which could predict cure at the end of therapy. This derived scoring system, the ATLAS score, was able to categorize CDI patients into 11 distinct categories and was able to predict clinical cure with a high degree of accuracy.

**Figure 2:**
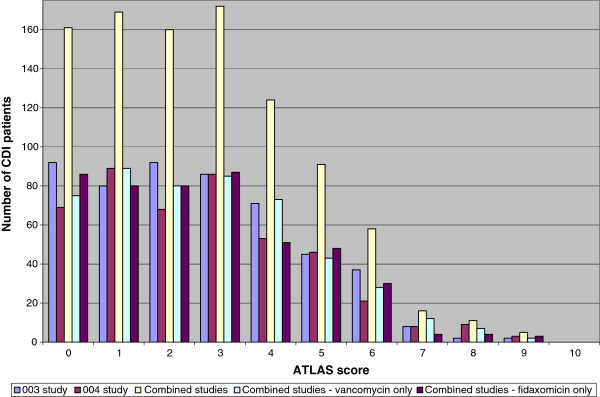
**Distribution of CDI patients in the two clinical trials (003 and 004), by ATLAS score.** The number of mITT patients in each of the groups who have available values for all analyzed variables: study 003 (n=515), study 004 (n=452), combined studies (n=967).

Several issues concerning these analyses should be mentioned. Firstly, the two databases used for the analyses were phase 3 clinical trials, which excluded extremely ill CDI patients. As per the study protocol, patients were excluded if they had “life-threatening or fulminant CDAD” (WBC >30 × 10^9^/L; temperature >40°C or evidence of hypotension and septic shock, peritoneal signs or significant dehydration), toxic megacolon, or were likely to die within 72 hours of study enrollment [4]. Therefore, there is an under-representation of CDI patients in the upper extremes of the score values. The distribution of patients, by ATLAS score, in the two clinical trial databases (Figure 2) may not represent all CDI patient populations in all healthcare systems. For instance, CDI patients in a specialty medical unit (e.g. transplant unit) or who manifest CDI while in an intensive care unit (ICU) may not conform to the prediction scheme, even though immunocompromised patients and ICU patients were enrolled into the two analyzed studies, if they were otherwise eligible. Secondly, other clinical or laboratory variables measured at the time of CDI diagnosis but not included in this analysis, may also aid in the predictive model of clinical cure. However, we chose clinical variables which have been repeatedly demonstrated to be correlated with disease outcome. Inclusion of the infecting strain type (i.e. NAP1/027/BI) into the categorization scheme might increase the predictive ability, since infections with this strain have been shown to increase the chance of poor outcomes from CDI [23]. However, at the present time, typing of the infecting strain is not widely available, and other non-NAP1/027/BI hyper-virulent strain types have already been described and may continue to emerge in the future [3].

Thirdly, a different use of the clinical variables with other weighting schemes, might give a more accurate predictive model. The variations in factor weighting and combinations are virtually limitless, and multivariate regression analysis was not helpful in determining the weighting scheme, since many of the factors highly predictive of outcome (i.e. albumin, age, serum creatinine) are very highly correlated with each other and negated each other in the multivariate models. Fourthly, it should be noted that only clinical cure was assessed in these analyses. We did a preliminary analysis investigating the capacity of this scoring system to predict CDI recurrence at 28 days post-therapy, and the five-component ATLAS score performed poorly in this regard. Other combinations performed better for predicting CDI recurrence, and those analyses will be undertaken at a later time. Lastly, this scoring system worked well in predicting response to both fidaxomicin and to vancomycin. Since no patients received metronidazole in these 2 clinical studies, it remains unknown how the ATLAS score would perform in predicting cure rates with this agent. However, the ATLAS categorization of CDI patients may now allow post-hoc comparisons of CDI patients treated with vancomycin and metronidazole in other databases in order to examine if there is truly a difference in outcome with these two agents in sub-groups of patients with specific scores.

## Conclusion

In conclusion, a combination of five simple clinical and laboratory variables measured at the time of CDI diagnosis, combined into an 11-category scoring system (ATLAS), seems to be able to accurately predict treatment response to CDI therapy by either vancomycin or fidaxomicin. The ATLAS scoring system may be useful to stratify CDI patients in order to prospectively evaluate and compare CDI therapies among patient categories, to choose therapies for patient sub-groups to maximize cure rates, to categorize patients in different CDI therapeutic studies in order to allow between-study comparisons of patient groups, and to stratify patients upon entry into CDI therapeutic trials.

## Competing interests

Dr. M. Miller was an advisory board member for Optimer Pharmaceuticals, Novartis, Sanofi and Merck and received research support from Optimer Pharmaceuticals. Dr. T. Louie received research support from Cubist, Cempra, Actelion and Optimer Pharmaceuticals and was a paid speaker for Optimer Pharmaceuticals. Dr. K. Mullane received research support and travel support for scientific presentations from Optimer Pharmaceuticals. Dr. K. Weiss received research support from Novartis, Merck and Optimer Pharmaceuticals and was a paid speaker for Optimer Pharmaceuticals. Dr. A. Lentnek and Dr. Y. Golan received research support from Optimer Pharmaceuticals. Y. Kean was an employee of Optimer Pharmaceuticals from 2010 to 2012, and Dr. P. Sears is currently an employee of Optimer Pharmaceuticals.

## Authors’ contributions

All authors have been substantially involved in all stages of the conception and design of this study and in the analysis and interpretation of the data. All authors have been involved in drafting the manuscript, revising it critically for its content and all have seen the submitted manuscript and have given final approval of the version to be published.

## Pre-publication history

The pre-publication history for this paper can be accessed here:

http://www.biomedcentral.com/1471-2334/13/148/prepub
